# Supplementary figures and images for: Mechanistic insights into coordinated var transcriptional switching in malaria parasites
Source: EMBO J. 2026 Mar 18;45(8):2614–37. doi: 10.1038/s44318-026-00751-x (PMC13083940; doi:10.1038/s44318-026-00751-x)

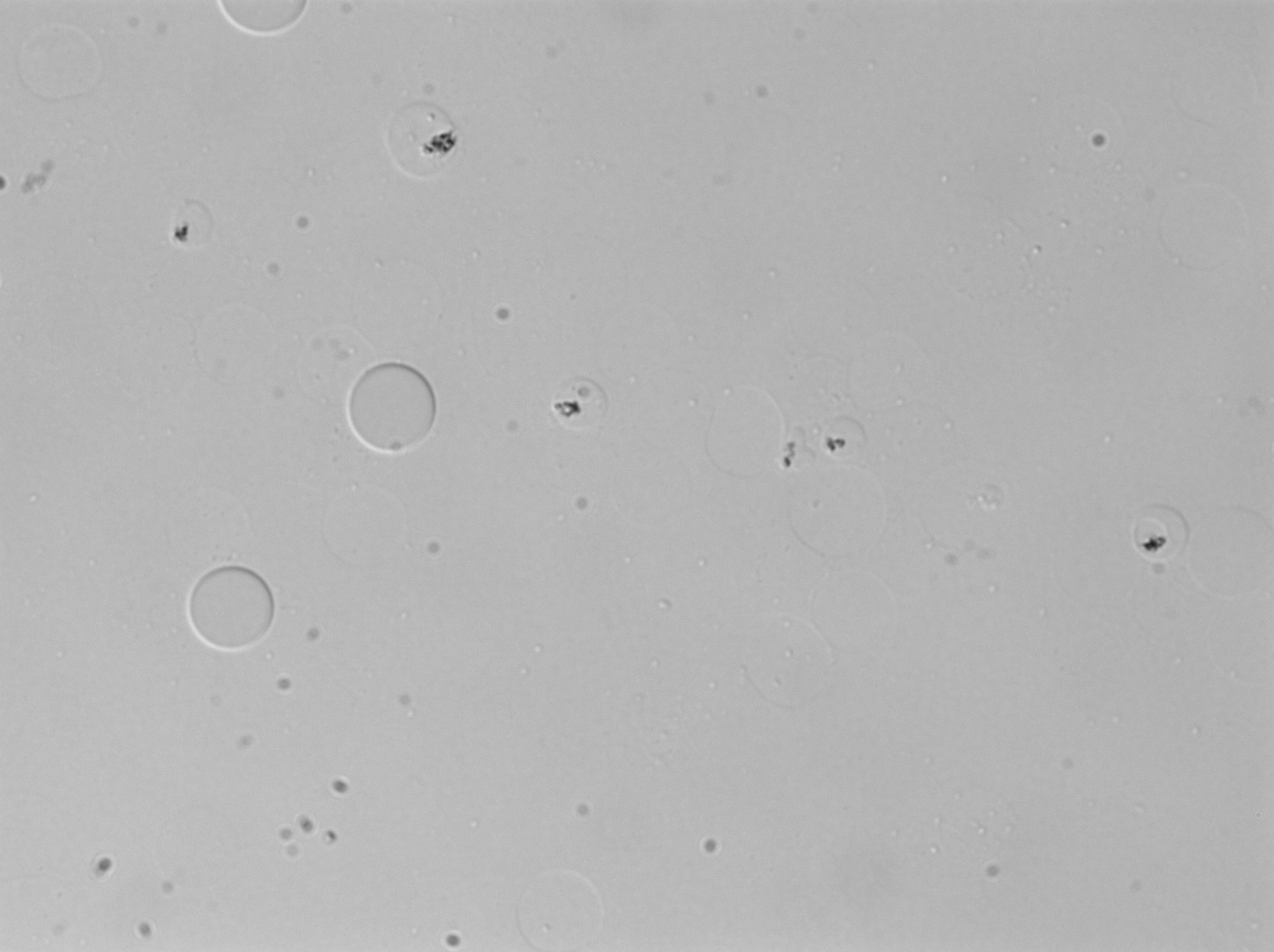

Supplement: Supplementary file 10 — Source data Fig. 5 [file 44318_2026_751_MOESM10_ESM.zip › Figure 5/5G/Original Images/3BKO_mNG.tif]

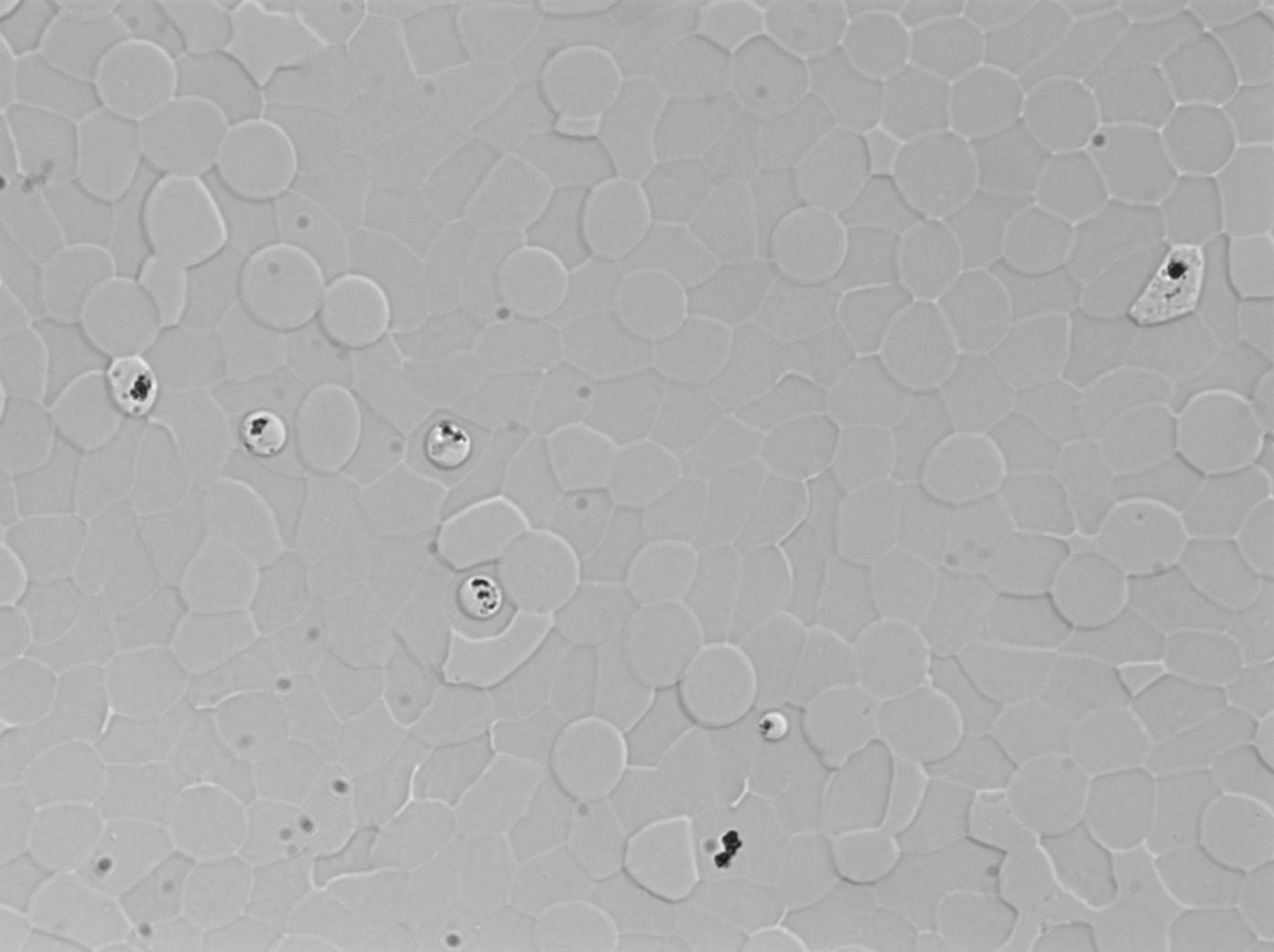

Supplement: Supplementary file 10 — Source data Fig. 5 [file 44318_2026_751_MOESM10_ESM.zip › Figure 5/5G/Original Images/mNG.tif]

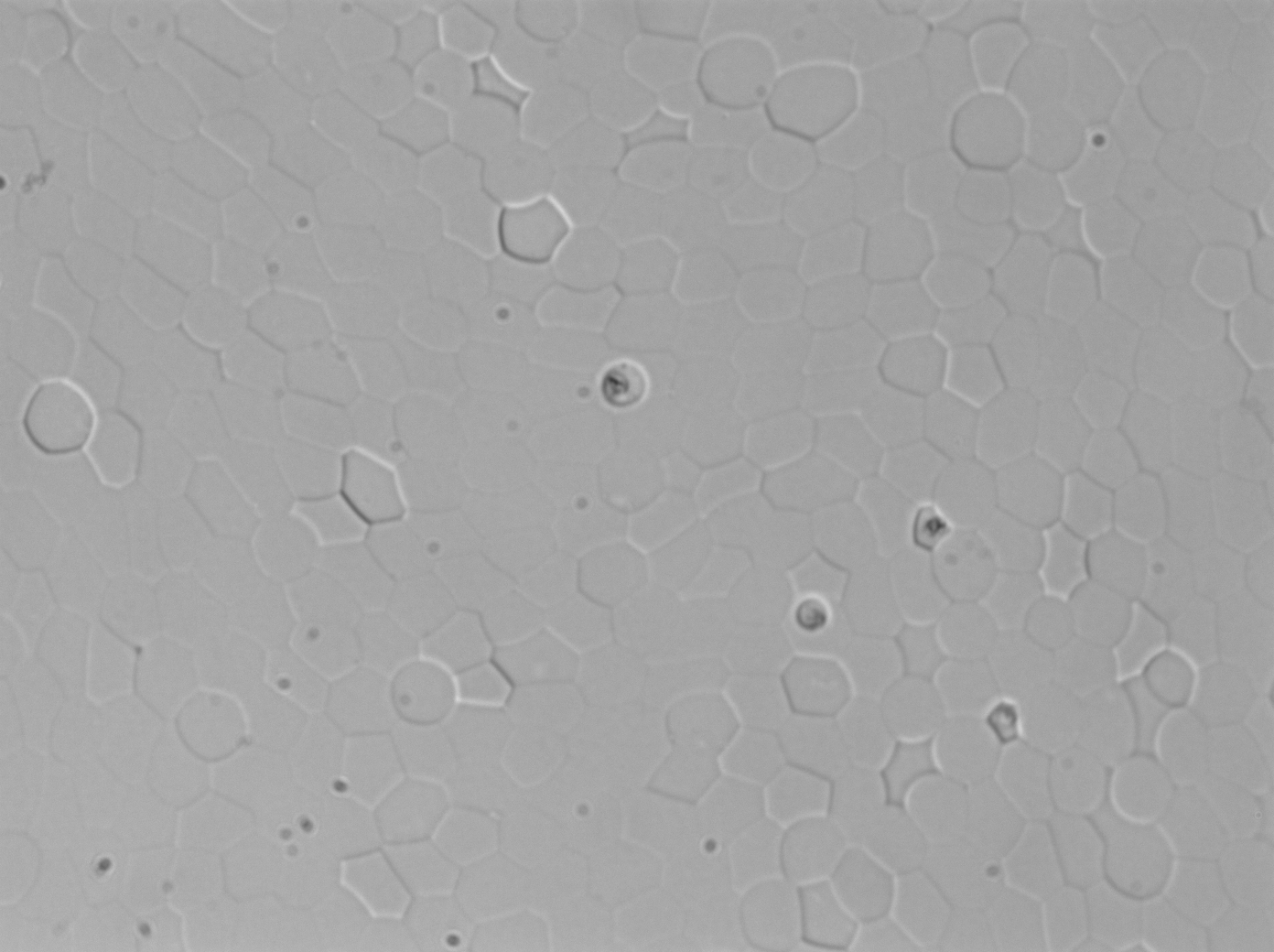

Supplement: Supplementary file 10 — Source data Fig. 5 [file 44318_2026_751_MOESM10_ESM.zip › Figure 5/5G/Original Images/mNG-BSD.tif]

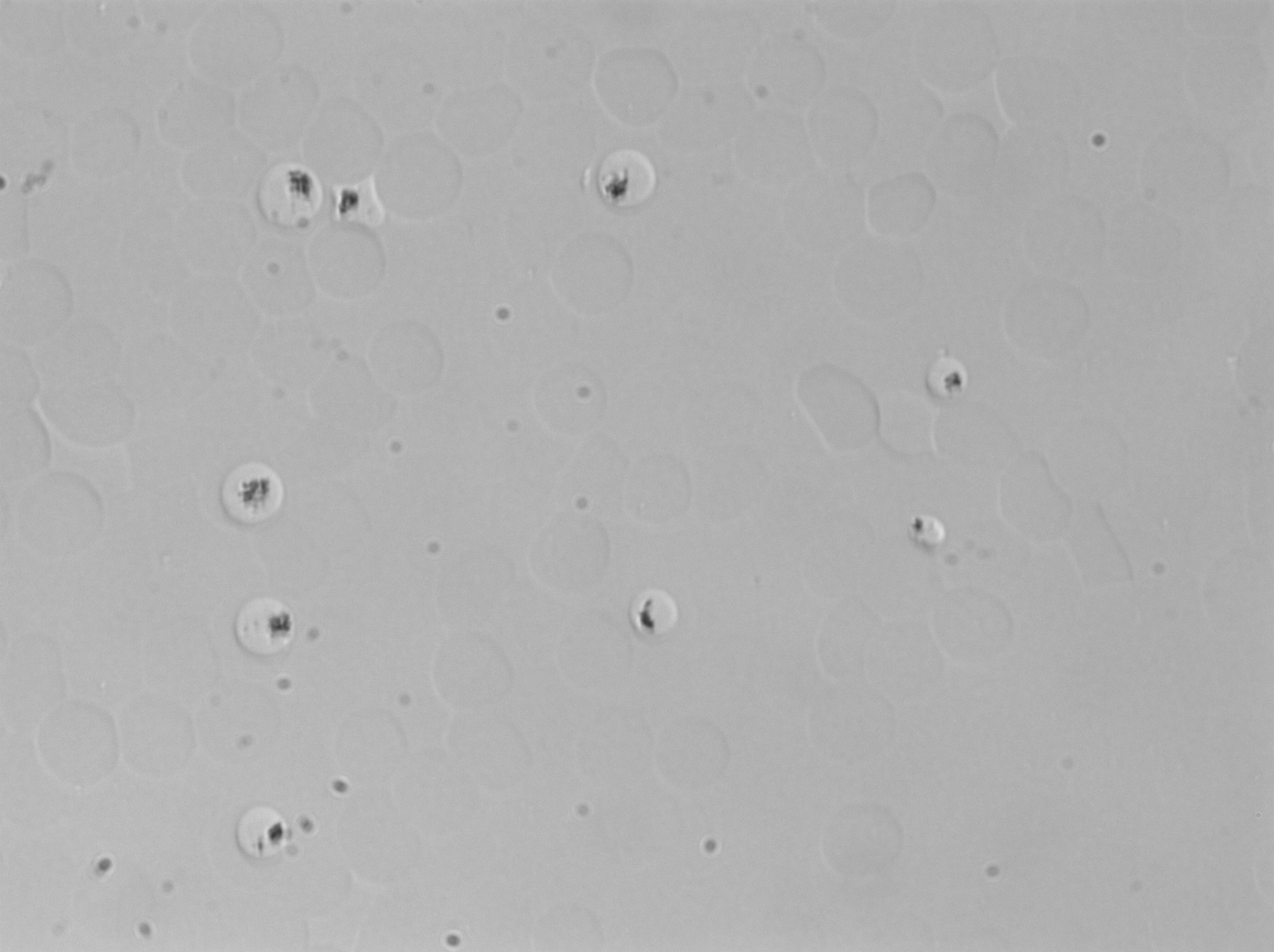

Supplement: Supplementary file 10 — Source data Fig. 5 [file 44318_2026_751_MOESM10_ESM.zip › Figure 5/5G/Original Images/wildtype.tif]

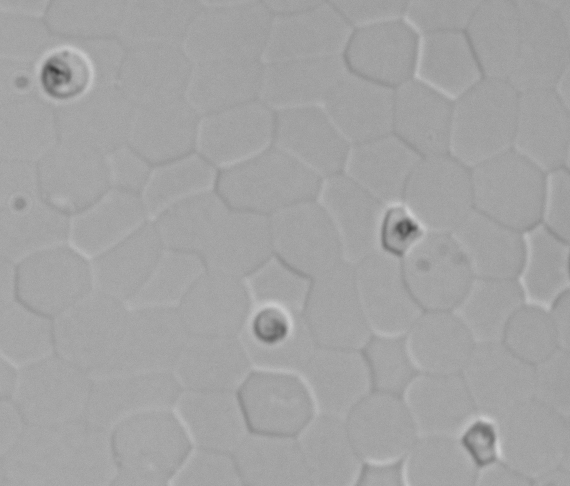

Supplement: Supplementary file 10 — Source data Fig. 5 [file 44318_2026_751_MOESM10_ESM.zip › Figure 5/5G/Cut Images/mNg-BSD/Ng FcuBsdDIC.tif]

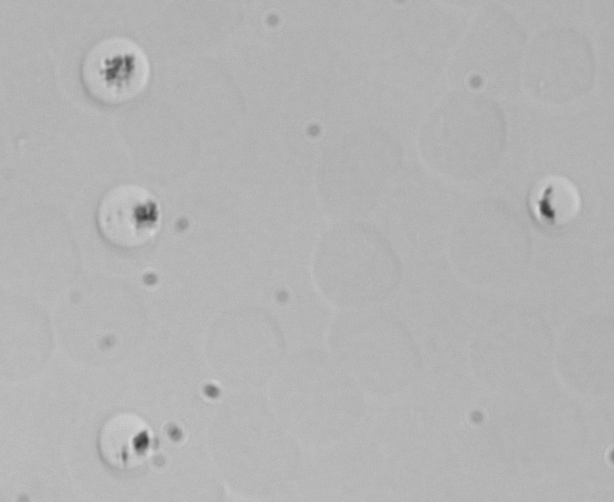

Supplement: Supplementary file 10 — Source data Fig. 5 [file 44318_2026_751_MOESM10_ESM.zip › Figure 5/5G/Cut Images/wildtype/WT A9DIC.tif]

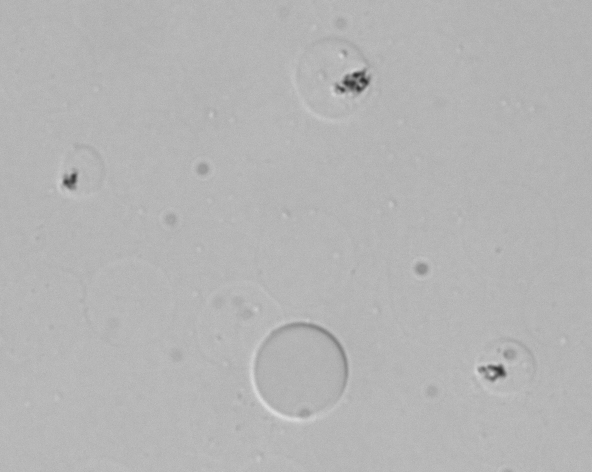

Supplement: Supplementary file 10 — Source data Fig. 5 [file 44318_2026_751_MOESM10_ESM.zip › Figure 5/5G/Cut Images/3BKO-mNg/3B NgDIC.tif]

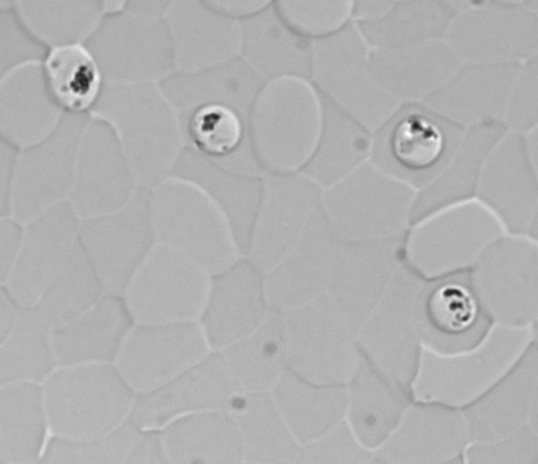

Supplement: Supplementary file 10 — Source data Fig. 5 [file 44318_2026_751_MOESM10_ESM.zip › Figure 5/5G/Cut Images/mNg /Ng uORFDIC.tif]
