# Supplementary material for: Mechanistic insights into coordinated var transcriptional switching in malaria parasites
Source: EMBO J. 2026 Mar 18;45(8):2614–37. doi: 10.1038/s44318-026-00751-x (PMC13083940; doi:10.1038/s44318-026-00751-x)
Supplement: Supplementary file 12 — Expanded View Figures [file 44318_2026_751_MOESM12_ESM.pdf]

Expanded View Figures

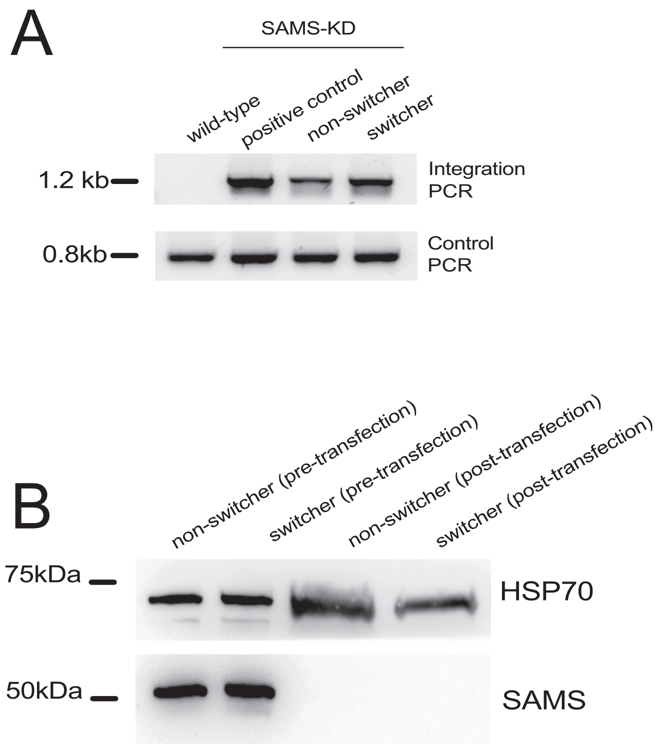

Figure EV1. Validation of SAMS-KD parasite lines.

(**A**) PCR validation confirming correct integration into the target locus. A wild-type, non-transfected line was used as a negative control, and a previously published SAMS-KD line (Harris et al, 2023) served as a positive control. Primer sequences are provided in Dataset EV3. (**B**) Western blot analysis using anti-SAMS and anti-HSP70 antibodies. Protein extracts were prepared from late-stage parasites of a wild-type non-switcher clone and a switcher clone, both before and after transfection with the SAMS-KD construct.

© The Author(s)

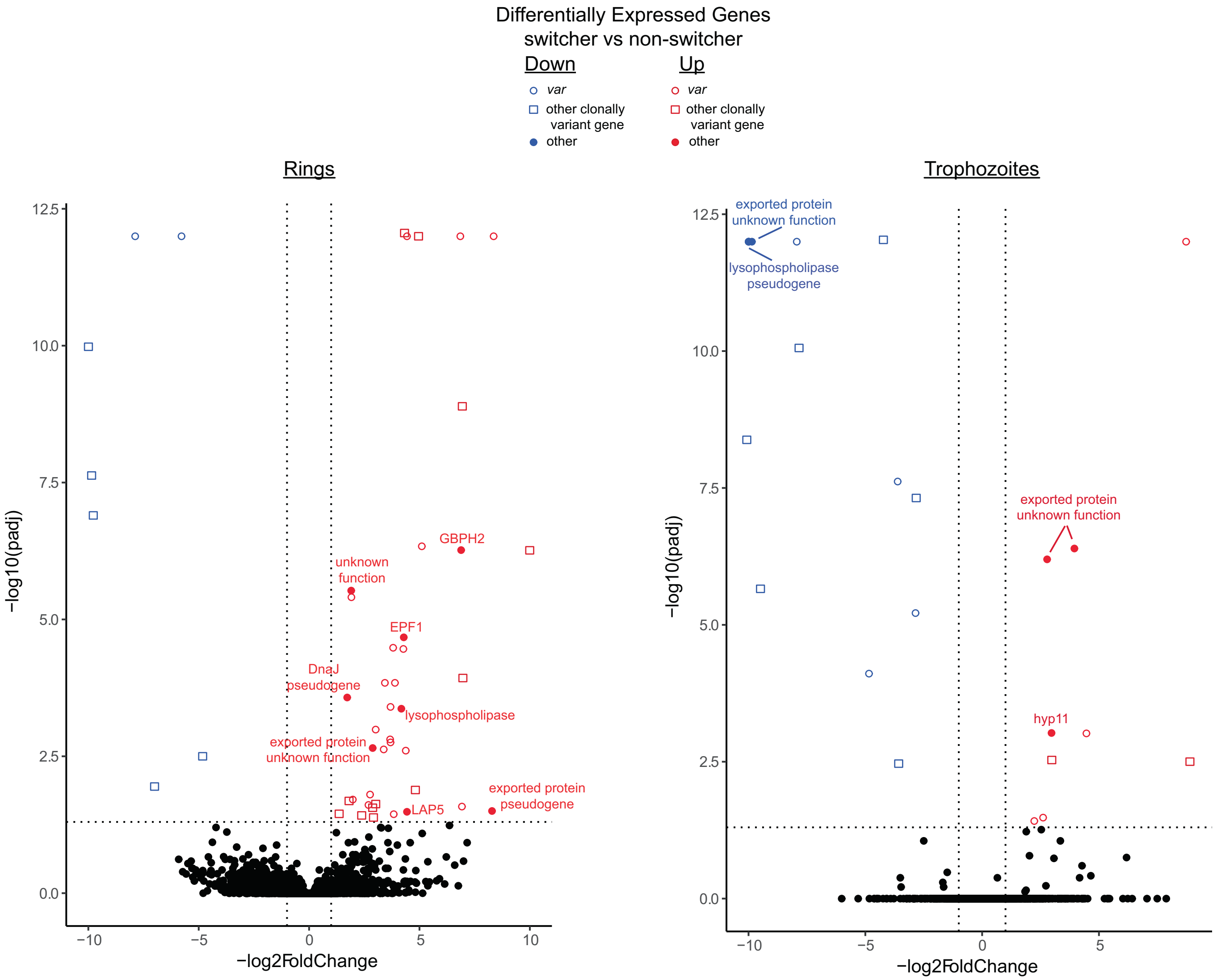

Figure EV2. Differential gene expression in switcher vs non-switcher lines determined by RNA-Seq.

Volcano plots displaying differential expression comparing the transcriptomes of switcher and non-switcher parasite populations. Upregulated genes in the switcher line are shown in red while downregulated genes are in blue. *var* genes are displayed as open circles while non-*var* genes from other clonally variant gene families are shown in open boxes. Other genes are labeled with the name of the encoded protein. Full annotation numbers for all genes are included in Dataset EV1. Comparison of ring-stage parasites are shown in the left panel and trophozoites are shown on the right. Differential expressional analysis was performed using DESeq2 (v.1.36) with a false discovery rate cutoff of <0.05. Comparison was performed with two independent replicate transcriptomes for each population. Differentially expressed genes were defined as having a log2foldchange of at least 2.00 and an adjusted p value of less than 0.05.

© The Author(s)

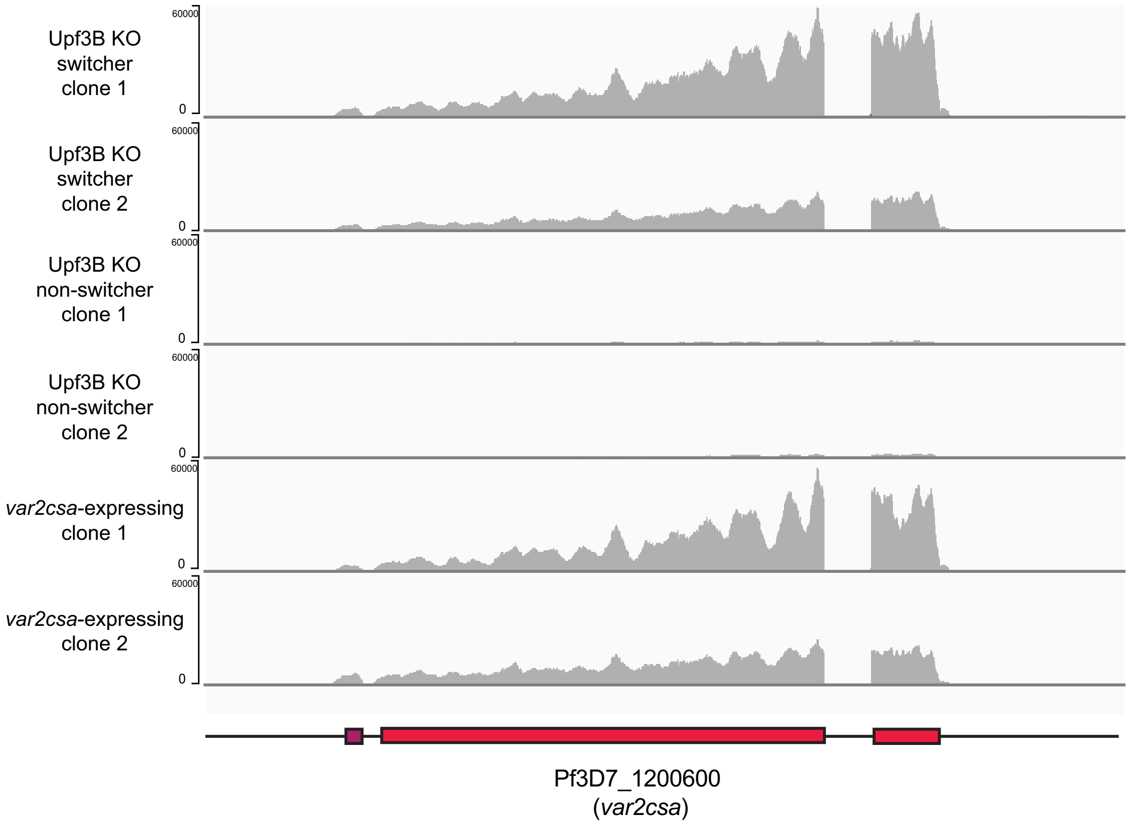

Figure EV3. Expression of *var2csa* in wild-type and Upf3B knockout lines detected by RNA-seq.

RNAseq profiles for the *var2csa* locus in either wild-type lines (bottom 2 profiles) or lines in which the *Pfupf3B* gene has been disrupted in either a switcher (top 2 profiles) or non-switcher (middle two profiles) background. Reads were normalized by downsampling. Each profile represents an independent biological replicate.

 © The Author(s)

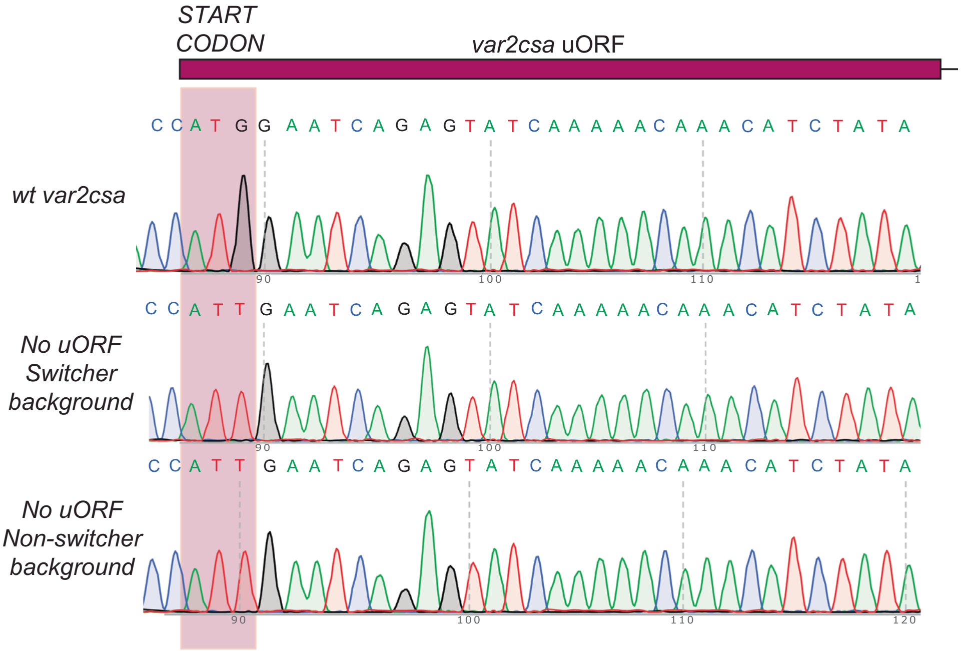

Figure EV4. Validation of uORF starting codon modification.

Sanger sequencing confirming the modification of the *var2csa* uORF start codon from ATG (methionine) to ATT (isoleucine) in both switcher and non-switcher backgrounds. A wild-type, non-transfected line is included as a control. The purple box highlights the three bases encoding the start codon.

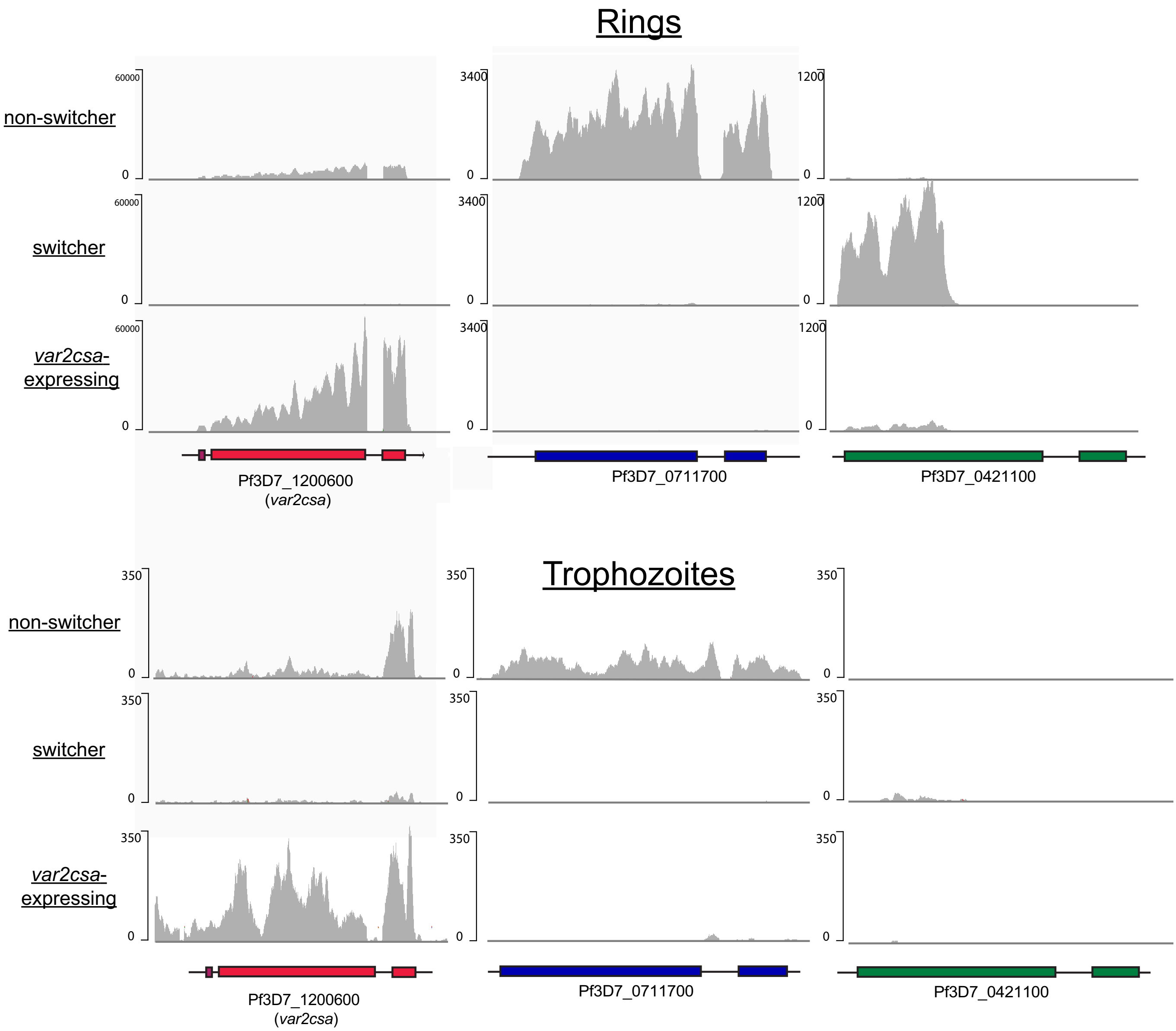

Figure EV5. Expression of dominant *var* gene in switcher, non-switcher and *var2csa* expressing lines detected by RNA-seq.

RNAseq profiles for the dominant *var* gene in both rings and trophozoites for a non-switcher (top), switcher (middle) and *var2csa* expressing line. Note that multimapping of reads was not allowed, thus for Pf3D7_0421100, only reads mapping to the 5′ end of the gene could be unambiguously mapped and are displayed. Reads mapping to the 3′ end of the gene were discarded due to near complete sequence identity elsewhere in the genome, thus preventing unambiguous mapping. Reads were normalized by down-sampling.

 © The Author(s)
